# Intravascular oxygen sensors with novel applications for bedside respiratory monitoring

**DOI:** 10.1111/anae.13745

**Published:** 2017-01-02

**Authors:** F. Formenti, A. D. Farmery

**Affiliations:** ^1^ Nuffield Division of AnaestheticsUniversity of OxfordOxfordUK; ^2^ Faculty of Life Sciences and MedicineKing's College LondonLondonUK

**Keywords:** ARDS ventilator management, dynamic arterial oxygen monitoring, fast optical oxygen sensor, respiratory arterial oxygen oscillations, ventilatory settings atelectasis

## Abstract

Measurement allows us to quantify various parameters and variables in natural systems. In addition, by measuring the effect by which a perturbation of one part of the system influences the system as a whole, insights into the functional mechanisms of the system can be inferred. Clinical monitoring has a different role to that of scientific measurement. Monitoring describes measurements whose prime purpose is not to give insights into underlying mechanisms, but to provide information to ‘warn’ of imminent events. What is often more important is the description of trends in measured variables. In this article, we give some examples ‐ focussed around oxygen sensors ‐ of how new sensors can make important measurements and might in the future contribute to improved clinical management.

## Measurement and monitoring

Measurement allows us to quantify various parameters and variables in natural systems. In addition, by measuring the effect by which a perturbation of one part of the system influences the system as a whole, insights into the functional mechanisms of the system can be inferred. Measurement is therefore the toolkit of science.

Clinical monitoring has a different role to that of scientific measurement. Monitoring describes measurements whose prime purpose is not to give insights into underlying mechanisms, but to provide information to ‘warn’ of imminent events. In general, the accuracy of data from clinical monitors is usually less important than those of similar scientific instruments. This is mostly because patient variables almost always have more intrinsic variability, so in a measurement snapshot, we have no way of knowing, a priori, whether the measured value deviates from an expected result because of measurement inaccuracy, measurement imprecision, patient variability or patient abnormality. What is often more important is the description of trends in measured variables. It is axiomatic that trends become more readily apparent if the variance attributable to the instrument is minimum (i.e. the measurements are precise). For this reason, although clinical monitors need not match the accuracy of similar scientific instruments, they should have a high degree of precision.

## Measuring the important vs. measuring the measurable

Anaesthesia and critical care have seen monitoring fashions come and go. In these environments, we tend to want to believe in the importance of the variables we can measure, but do not give much consideration to those we cannot. This problem is widely acknowledged in many areas of life. The US Defence Secretary and military data analyst at the time of the Vietnam war, Robert S McNamara, famously stated that ‘We must avoid making what is measureable important and find ways to make the important measurable.’

As clinicians, we also build our understanding of the basis of disordered physiology around what we have in our measurement toolkit. So in a real sense, what we can measure dictates what models we formulate, and this can be a problem for anyone wanting to break free of orthodoxy. As Thomas Kuhn put it ‘observations are expected to be interpreted in the context of the accepted paradigm thus restricting the peripheral vision of the researchers, since they reject the observations if they do not fit the model.’

In this article, we give some examples of how new sensors can make important measurements that might challenge the accepted wisdom, and might in the future contribute to improved clinical management.

## Dynamic blood gas analysis

Our understanding of the pulsatile nature of arterial blood flow in a distensible arterial tree means that we can fully appreciate the added value of monitoring arterial blood pressure dynamically by arterial cannulation and a rapid response transducer system. All would agree that identifying systolic and diastolic pressures, the waveform and even its power spectrum provide important information. No one would imagine that monitoring the mean arterial pressure alone would be just as valuable. Yet, when it comes to blood gas analysis, intermittent sampling to provide a spot reading of mean PaO_2_ is regarded as acceptable because it fits with the notion that PaO_2_ is not a dynamic variable, which is consistent with the simple ‘balloon on a straw’ model of the lung, based on continuous ventilation and perfusion principles on which we were brought up 1. What if, under certain circumstances, PaO_2_ was as dynamic as an invasive arterial blood pressure signal? What would this tell us? How would we have to rethink our models and understanding? Would we be interested?

Dynamic within‐breath oscillations in PaO_2_ have been reported in animal and human studies for the past five decades 2, 3, 4, 5, 6. The early studies attracted little attention, but Williams et al. in 2000 7, followed by Baumgardner et al. in 2002 2, noting that these oscillations were more marked in the injured or atelectatic lung, hypothesised that dynamic PaO_2_ signals could have diagnostic potential. But this would require us to revise our models of gas exchange.

At this time, gas exchange efficiency (which might be simplified as the degree to which ventilation and perfusion are directed away from the ideal alveolus, and towards the alveolar dead‐space or shunt compartments, respectively) was considered to be constant in the short term. Although this might vary as the patient's condition changed over hours or days, it was considered to be a parameter (a quasi constant) rather than a dynamic variable.

The proposition that in acute lung injury, PaO_2_ oscillations could be caused by the development of atelectasis of dependent regions at end‐expiration and subsequent recruitment during each inspiration (cyclical recruitment, CR) was modelled by Whiteley et al. 8 and shown to be consistent with experimental observations. Moreover, evidence in support of the existence of CR had already been demonstrated in animal models of lung injury by other techniques such as rapid sequence CT imaging by Hedenstierna's group 9 and by subpleural microscopy by Nieman's group 10. The monitoring of PaO_2_ oscillations might therefore be a useful alternative indicator of this process, and has the benefit of being achievable at the bedside.

The clinical importance of this measurement is that CR of atelectasis is thought to play a role in the development of ventilator‐induced lung injury, as the shear stresses imparted by the alveolar expansion generate a cellular inflammatory response and so‐called atelectotrauma 11, 12. Such an inflammatory trigger may even be the driver to a systemic inflammatory response 13. If CR can be detected by rapid continuous PaO_2_ measurement at the bedside, it has the potential to influence and personalise treatment. However, controversy exists with respect to the relative contributions of CR and overdistension of normally aerated and hyperinflated regions of lung in this heterogeneous condition. Kavanagh showed in a rat model of ARDS/ventilator‐induced lung injury that the major alveolar inflammatory response was localised not in atelectatic regions but in non‐dependent aerated regions. Distal airway injury occurred in both dependent and non‐dependent regions alike 14. Similarly, Terragni et al. showed in human patients imaged with CT, that those in whom tidal ventilation was predominantly distributed to hyperaerated lung regions (and who also had larger areas of atelectasis) showed a greater inflammatory response – as measured by pulmonary cytokine concentrations – than those in whom tidal ventilation was predominantly distributed to normally aerated lung (and who also had smaller areas of atelectasis) 15. Neither of these observations disprove the potential of CR to induce local and distant injury, but it is clear that hyperinflation elsewhere is potentially equally injurious and often co‐existent.

## The technology of photonic oxygen sensing

Traditionally, oxygen in the blood phase has been measured by Clarke‐type electrodes as are found in bench blood gas analysers. Electrochemical methods such as this are difficult to deploy in vivo because of problems of noise, drift and fouling of the sensing element of the electrode by proteins and fibrin micro‐aggregates. In recent years, optical or photonic sensing technology has developed rapidly in a number of areas including oxygen sensing. The Paratrend 7, a multiparameter intravascular sensor using an early embodiment of this technology, was developed by Diametrix Medical (High Wycombe, UK) in the 1990s, but failed to gain a market because, with its slow response time (> 70 s), it was thought to offer little more than standard intermittent blood gas sampling or pulse oximetry. Rapid sensors designed for industrial use have been used extensively in animal research, but no clinically licensed intravascular sensor yet exists. Just as an arterial blood pressure transducer requires a rapid response in order to faithfully measure the arterial waveform, so too does any potential dynamic PaO_2_ sensor, especially if required to track high respiratory rates 16.

**Figure 1:**
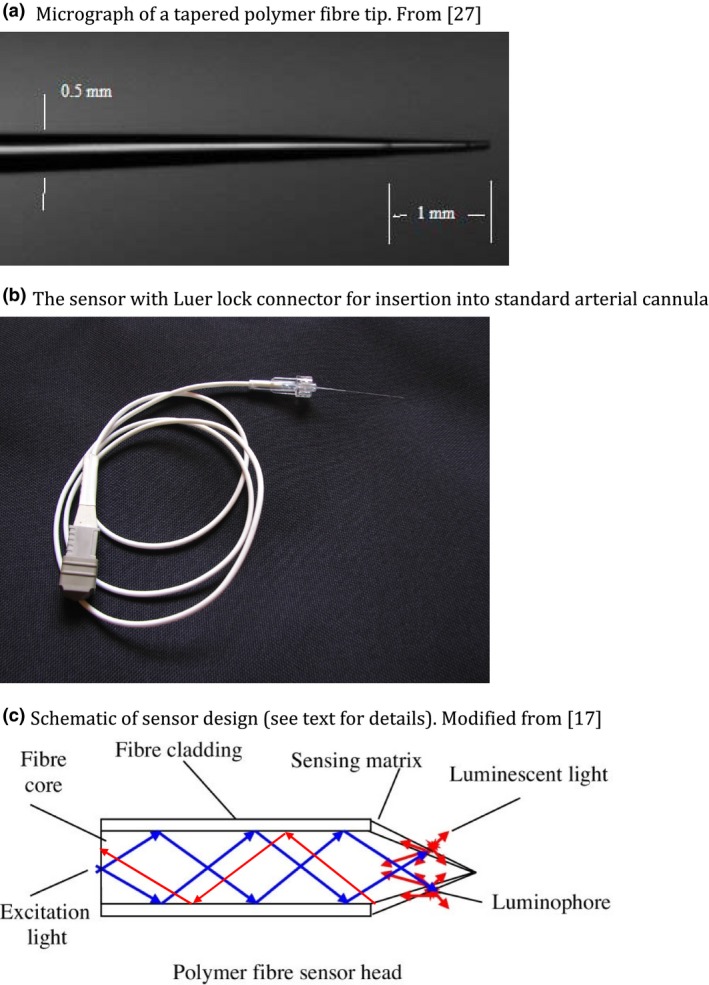
Micrograph of a tapered polymer fibre tip. From 27, the sensor with Luer lock connector for insertion into standard arterial cannula. Schematic of sensor design (see text for details). Modified from 17 *Correction added on 6 January 2017, after first online publication on 3 January 2017. Figure 1c has been replaced due to some red arrows appearing outside the boundaries of the figure.

Photonic oxygen sensors exploit the property of molecular oxygen to quench the quantal luminescence emissions of certain excited luminophores. These luminescent dyes can be doped onto the tip of an optical fibre, which can be placed in a standard arterial cannula. Pulsed high‐energy light can be launched through the fibre waveguide to interact with the fluorophore at the distal tip as shown in Fig. 1. This excitation promotes electrons within the sensing luminophore to higher energy levels and as they return to ground state they emit discrete photon packages at a lower energy. Typically, the fluorescence decays away very rapidly with a time constant in the order of microseconds. The higher the ambient oxygen concentration, the greater the quenching of this process – due to energy transfer consequent on molecular collision – the shorter the decay time constant and the weaker the light intensity signal. The relationship between the intensity of the emitted fluorescence and the ambient oxygen concentration is given by the Stern–Volmer equation:

(1) $$\frac{I}{I_{0}}=\frac{1}{1+k_{SV}\left[O_{2}\right]}$$

where *I* is the light intensity, *I*_0_ is the light intensity in the absence of oxygen, *k*_*SV*_ is the Stern–Volmer constant and [O_2_] is the oxygen concentration in the sensor matrix.

The emitted light is transmitted back down the fibre to a spectrophotometer where it can be measured.

## Challenges to sensor design

There are a number of challenges in the design of these optical oxygen sensors. First, for clinical use they must be made of inert unbreakable material with a non‐toxic luminophore. Polymer fibres and platinum porphyrin‐based luminophores fulfil this need. Such fibres can be 300–500 μm in diameter, which allows them to be inserted into a standard arterial cannula (see Fig. 1a,b). However, for such small sensors, the amount of luminophore that can be accommodated on the blunt tip end, and the amount of light that can be launched, is limited, thereby limiting the signal‐to‐noise ratio. The sensor design from 17 shown in Fig. 1c overcomes this limitation to some extent by using a tapered tip design. Here, excitation light is launched down the plastic fibre core whose refractive index is greater than that of the cladding layer. As a result, the light undergoes total internal reflection (blue arrows in Fig. 1c). On nearing the tip, however, the fibre is tapered in such a way as to distort the geometry of the reflective interface such that transmission rather than internal reflection occurs and the light now leaks from the core into the cladding. This taper provides a larger surface area for interaction between the excitation light and the luminophore, which is embedded in a polymer matrix over the tapered region. Much of the luminescent light (illustrated by red arrows in Fig. 1c) is transmitted back down the taper cladding and then into the fibre core by a reverse of the same process described.

One potential problem is that a small fraction of the fluorescent light, rather than returning directly back down the fibre, is emitted into the arterial blood and is scattered by red cells. Some of this scattered light may be retransmitted back down the fibre, but this depends of the nature of the blood in which it sits (i.e. the haemoglobin concentration and its saturation). Consequently, the intensity of the fluorescent light, which is the variable of interest in Equation (1), may be affected by factors other than the PaO_2_ itself. To overcome this problem, rather than measuring light intensity itself, Farmery et al. have adopted the technique of measuring the time constant of the quantal luminescence emissions themselves, which are solely a function of the oxygen concentration (or PaO_2_), and therefore immune from influences from the sensed medium.

**Figure 2:**
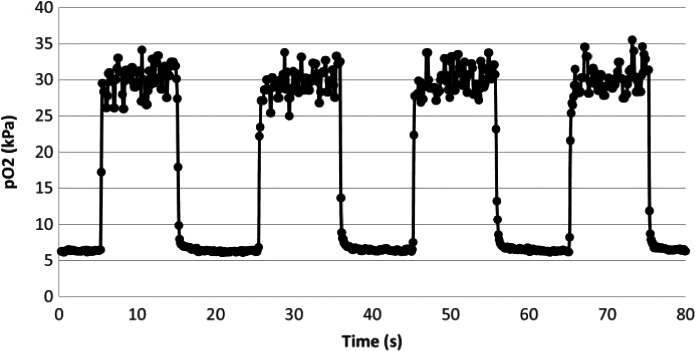
Response of fibre‐optic oxygen sensor to near step‐changes in PO_2_ in the liquid phase. Sampling points are at 10 Hz, that is, 100 ms intervals. From 17

The requirements for both speed and sensitivity of the sensor are seemingly difficult to reconcile. On the one hand, in order for the luminophore to ‘see’ any oxygen at all, we need to embed it in a matrix (the polymer glue that sticks it to the sensor tip), which has a high oxygen solubility. On the other hand, if the sensor matrix has a high oxygen solubility, it will act as a large oxygen reservoir which will slowly fill and slowly empty as the PaO_2_ rapidly steps up and down. Consequently, this would give a very slow signal response. These opposing requirements and properties have been reconciled by the use of blends of copolymers with large pendant side chains 17, which have a higher intrinsic diffusivity, and by the addition of novel nanostructures into the matrix which increase the diffusivity and allow oxygen to remain at least partly in the gas phase as it enters and leaves the sensing matrix (rather than dissolving in the polymer and forming a reservoir). As a result, the sensors are capable of tracking very rapid changes in PO_2_ in the liquid phase, as shown in Fig. 2. In this study, a sensor was placed in an extracorporeal circuit comprising two independent circulations with high (30 kPa) and low PO2 (5 kPa). Flow from each of these circulations was directed alternately over the sensor by actuation of rapid solenoid switch valves. It is not possible to quantify the response time of the sensor with accuracy because the flow switch‐over process itself is the rate limiting step, but it is evident that even despite this, the rise time was of the order of 100 ms. In the gas phase, these sensors can have response times of less than 50 ms 18.

## Observations of dynamic oxygen signals in the normal and injured lung – ‘normality’

Cyclical variations in alveolar gas composition have long been understood to occur in normal physiology. DuBois et al. predicted and modelled oscillations in alveolar PCO_2_ (and by inference also PO_2_) throughout the respiratory cycle in the normal lung 19, and Band and Wolff demonstrated within‐breath oscillations in arterial pH (a proxy for PCO_2_) in normal man 20. So if oscillations in PaO_2_ are a feature of normal physiology, how are they different from those generated by cyclical recruitment in the injured lung?

**Figure 3:**
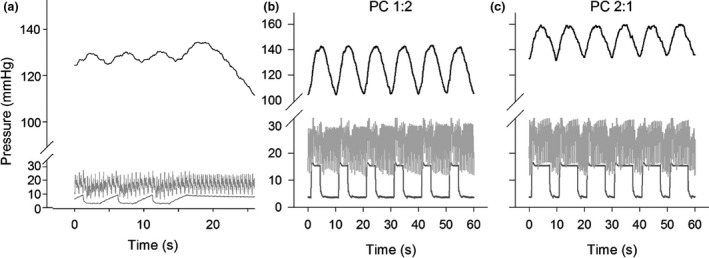
Representative continuous measurements of PaO_2_ (top, black), pulmonary artery pressure (middle, light grey) and airway pressure (bottom, dark grey) in a healthy pig, presented as a function of time. (a) Ventilation was delivered in volume control mode, I:E ratio was 1:1, respiratory rate was 12 min^−1^ and airway pressure ranged from positive end‐expiratory pressure of 5 cmH_2_O to end‐inspiratory pressure of 10 cmH_2_O. (b) and (c) Ventilation was delivered in pressure control mode (PC) with I:E ratios of 1:2 and 2:1, respectively. Respiratory rate was 6 min^−1^, and airway pressure ranged from positive end‐expiratory pressure of 5 cmH_2_O to end‐inspiratory pressure of 15 cmH_2_O. Data from experiments detailed in 21.

Figure 3 shows oscillations in PaO_2_ in an anaesthetised ventilated pig without lung injury, that is, with as close to normal physiology as is possible under the experimental conditions 21. Here, oscillations in PaO_2_ corresponding to the respiratory cycle can clearly be seen. Note that in apnoea at end‐inspiration (Fig. 3a), the decline in PaO_2_ is quite linear when haemoglobin is almost saturated (PaO_2_ > 100 mmHg), but although not shown here, the process becomes non‐linear as the inflection point of the oxyhaemoglobin dissociation curve is approached (PaO_2_ < 100 mmHg).

The amplitude of the tidal PaO_2_ oscillation, and the rate of fall in apnoea can be predicted by a simple mass balance model proposed (for CO_2_ oscillations) by Dubois et al. 19 and Edwards et al. 22. In essence, this is formulated from the assumption that the rate of change in oxygen concentration in the alveolar compartment is the algebraic sum of the rate at which it is added (or removed) by ventilation, and the rate of uptake by the pulmonary circulation. During apnoea, the rate of decline of PaO_2_, d(PaO_2_)/dt is given by the following reduced form of the general equation:

(2) $$\frac{{dP}_{a(t)}}{dt}=P_{B}.\frac{{\dot{v}}_{O_{2}}}{V_{A}+V_{T}}$$

where *P*_*B*_ is the barometric pressure, ${\dot{v}}_{O_{2}}$ is the rate of oxygen uptake, *V*_*A*_ is the lung volume at end‐expiration and *V*_*T*_ is the tidal volume at the point of apnoea (i.e. at end‐inspiration *V*_*T*_=10 ml.kg^−1^ in Fig 3a).

The rates of apnoeic decline predicted by Equation (1) (based on independently measured values of ${\dot{v}}_{O_{2}}$ and *V*_*A*_) and those measured by the sensor show reasonable agreement over a range of lung volumes and oxygen uptake rates (unpublished results).

The peak to trough amplitude of the tidal oscillation can be predicted as a first approximation by assuming that it is given by the product of the rate of decline in expiration and the expiratory time. This can be determined from a modification of Equation (1), where *f* is the respiratory frequency and R_IE_ is the I:E ratio:

(3) $$\begin{array}{cc} \Delta {\text{PaO}}_{2}\; & =\;P_{B}\cdot \frac{{\dot{v}}_{O_{2}}}{V_{A}+V_{T}}\cdot \frac{1}{f(1+R_{IE})}\\ & =\text{rate of decline in apnoea}\times \frac{1}{f(1+R_{IE})} \end{array}$$

Figures 3a–c show continuous PaO_2_ recordings in a healthy pig under different ventilatory conditions. The observed peak‐to‐trough amplitudes of the tidal oscillations agree approximately with values predicted based on simple mass balance modelling from Equation (1).

Figures 3b and c show the mean PaO_2_ and the amplitude of respiratory PaO_2_ oscillations during mechanical ventilation with I:E of 1:2 (3b) and 2:1 (3c). The I:E of 2:1 was associated with greater mean PaO_2_ level, and smaller amplitude of PaO_2_ respiratory oscillations. Mean PaO_2_ increased from 113 ± 22 (I:E 1:2) to 127 ± 25 mmHg (I:E 2:1). The amplitude of the PaO_2_ oscillations decreased from 28 ± 6 (I:E 1:2) to 21 ± 5 mmHg (I:E 2:1).

## The injured lung

**Figure 4:**
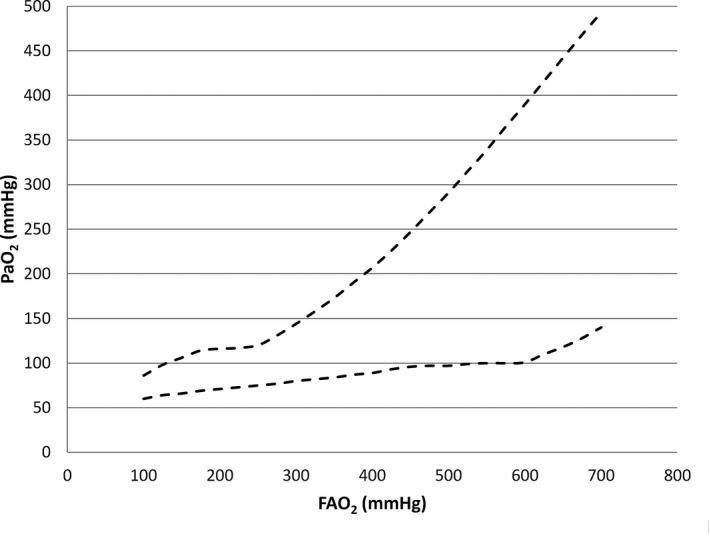
Predicted PaO_2_ oscillation peaks and troughs as a function of FAO2 for the modelled scenario where shunt fraction changes cyclically, breath by breath, from 30% at end‐expiration to 10% at peak inspiration. Dotted lines show the envelopes of the peaks and troughs of the oscillation.

It seems that certain defining qualitative and quantitative aspects of the respiratory PaO_2_ oscillations seen in ARDS/acute lung injury distinguish them from those that are part of normal physiology. In a saline lavage rabbit model of ARDS, Baumgardner 2 demonstrated intrabreath oscillations as wide as 400 mmHg (100 mmHg trough, 500 mmHg peak) with an F_I_O_2_ of 1.0, even using a relatively slow optical sensor, so clearly it is likely that ‘pathological oscillations’ might be distinguished from ‘physiological’ ones by magnitude alone, especially since the expected physiological magnitude can be calculated from estimates of oxygen consumption, functional residual capacity (FRC) and respiratory rate according to Equations (1) or (1). Secondly, Equation (1) tells us that the physiological oscillation is independent of the prevailing F_I_O_2_ or F_A_O_2_, since neither of these variables feature in it. On the other hand, if we propose that in ARDS/acute lung injury, the PaO_2_ oscillations are due to cyclical changes in shunt fraction resulting from cyclical recruitment/derecruitment, then theoretical modelling predicts that the PaO_2_ oscillation amplitude would vary as F_I_O_2_ (and hence F_A_O_2_) varies. This prediction is shown in Fig. 4, which shows a simulation of the effect of varying F_A_O_2_ on the peak and trough of a PaO_2_ oscillation in a scenario where shunt fraction is set to cycle from 30% at end‐expiration to 10% at end‐inspiration.

**Figure 5:**
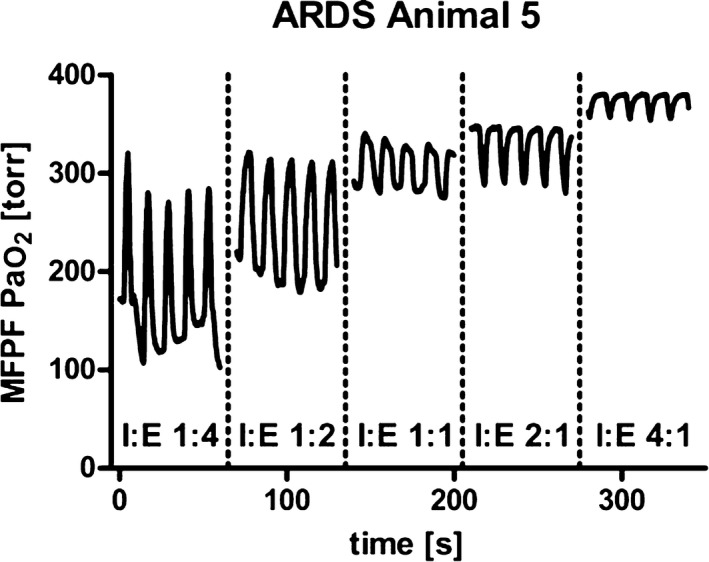
Example of continuous PaO2 measurement in experimental acute respiratory distress syndrome (ARDS). Different inspiration to expiration (I:E) ratios were studied during ongoing mechanical ventilation (from 25). MFPF, multi‐frequency phase fluorimetry.

In the injured lung, the PaO_2_ signal does not follow the predictions of Equations (1) and (1), and it is not easily predictable. It can also show considerable interindividual variation, which is not surprising given the variable nature of ARDS/acute lung injury. The signal may therefore provide a personalised insight into a particular patient's pathophysiology. The mechanisms underlying the observed PaO_2_ changes throughout the respiratory cycle have not been fully established, but work to relate intrabreath PaO_2_ oscillations, and dynamic gas exchange is ongoing in animal models of ARDS using rapid sequence CT imaging and PET imaging of pulmonary blood flow. Figure 5 shows the dynamic PaO_2_ signal in a piglet that has undergone repetitive washout of surfactant to simulate ARDS. The F_I_O_2_ was 1.0, the respiratory rate was relatively low (6 min^−1^) 23, the I:E ratio is initially 1:4, exaggerating intrabreath oscillations. Here, PaO_2_ oscillates between 100 mmHg and 300 mmHg, and the rising signal is synchronous with lung inflation. This phenomenon is consistent with the idea that cyclical recruitment and derecruitment might underlie the PaO_2_ oscillations, as confirmed by the changes in the shape of the PaO_2_ trace when I:E is inverted. This change is consistent with the possibility that the shorter expiratory time lessens the derecruitment effect and so moderates the PaO_2_ oscillation amplitude.

**Figure 6:**
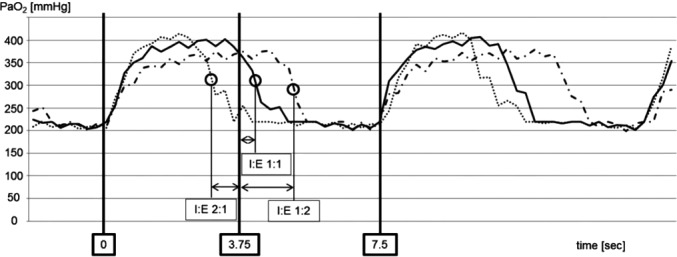
Example of inverted oxygen oscillations in a mechanically ventilated pig. The I:E of 1:2 is associated with a long PaO_2_ peak and short trough, and vice‐versa with the I:E of 2:1. Data from 24

In other instances, the signal can behave differently. Figure 6, from Bodenstein et al. 24 shows a record from a different animal, ventilated with F_I_O_2_ of 1.0, at 8 breaths.min^−1^.

The Pa/FI ratio in this case is not dissimilar from that shown in Fig. 5 (i.e. in both cases moderate gas exchange impairment is evident), yet in Fig. 6 the PaO_2_ signal has a seemingly paradoxical phase relationship with airway pressure, that is, PaO_2_ falls in inspiration and rises in expiration. In practice, the phase relationship is not easy to determine by eye because the raw PaO_2_ signal, measured in the ascending aorta in these experiments, is usually a couple of seconds delayed with respect to the airway pressure signal, but it can be inferred from the changes in the PaO_2_ signal in response to changes to the I:E ratio, which can be imposed from time to time. Perhaps counterintuitively at first, it appears that the PaO_2_ trace has a relatively long peak, and a short trough when the I:E ratio is 1:2. This trend is inverted when the I:E ratio is inverted to 2:1. In this second example, the effect of I:E reversal on the mean value of PaO_2_ and on the amplitude of PaO_2_ oscillations is not apparent, possibly because the pigs were not surfactant depleted, in contrast with the pigs studied in 25.

At the moment, the mechanism behind the paradoxical phase relationship is in part speculative, but it may involve redistribution of pulmonary blood flow due to pulmonary capillary compression in overstretched lung units, such that in inspiration ideal pulmonary capillary flow is reduced and shunt blood flow dominates. An analogous form of this phenomenon has been demonstrated with the worsening of arterial oxygenation following a recruitment manoeuvre in a sheep lavage model of acute lung injury, studied at a low time resolution with positron emission tomography 26.

## Conclusions

**Figure 7:**
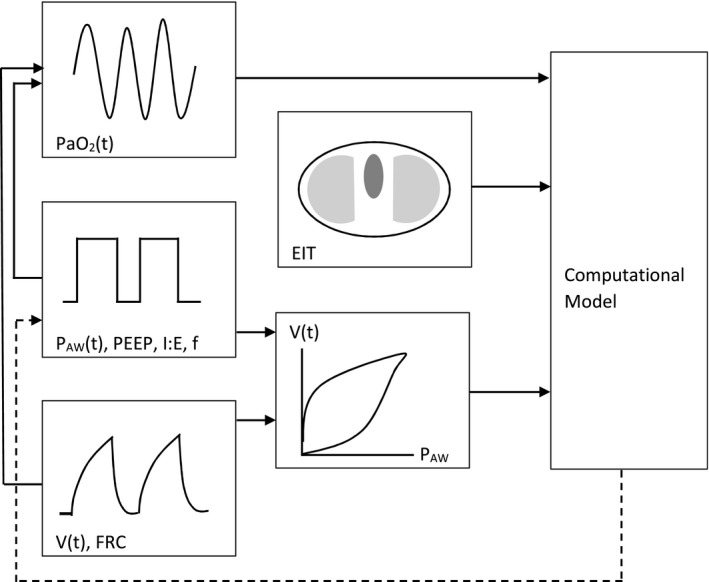
Potential multisource data input to computational model of individual patient lung dysfunction. Data from dynamic PaO_2_, and the effect of cyclic pressure and distension changes on the waveform and phase relationship of the signal, inform the model. Output from the model may then inform ventilator management.

Rapid PaO_2_ sensing by a small plastic fibre‐optic sensor capable of being inserted into a standard arterial cannula, has the potential to change the way clinicians understand disordered gas exchange in patients with lung injury. The condition is heterogeneous throughout the lung, and highly variable both between patients and within individual patients at different time points in their illness. Static blood gas analysis and assessment of PaO_2_/F_I_O_2_ ratio are too insensitive to characterise the complex and unique sets of circumstances that conspire to produce the observed gas exchange deficits in ARDS patients. Less likely still is a ‘one size fits all’ approach to ventilator management, based on routine bedside measurements, to benefit all patients. More work is needed to invert individual patients’ dynamic PaO_2_ data to create an individualised model of the prevailing dysfunction at any particular moment. The next challenge is to develop a computational model informed by an array of different sensing inputs from a number of sources, including dynamic PaO_2_, to provide the clinician with real‐time information on a patient's gas exchange efficiency and its responsiveness. This system might be informed by other data such as airway pressure, volume (and particularly the effect that cyclic changes in pressure and distension have on the dynamic PaO_2_ signal), FRC and information on spatial aeration from modalities such as electrical impedance tomography, as proposed in Fig. 7.

With guidance from dynamic PaO_2_ sensing, ventilator management might be tailored to minimise cyclical recruitment/derecruitment where it exists, or to avoid unnecessary and excessive PEEP and recruitment manoeuvres where these are shown to be ineffective. Dynamic PaO_2_ sensing also has the potential to detect when excessive pressure or distension of already aerated lung impairs blood flow to the ‘ideal alveolus’, allowing better informed choices of PEEP, respiratory rate, and I:E ratio.

We have come a long way from considering that ventilation and gas exchange are steady‐state continuous processes like those occurring at a fish's gills. The tools are being developed to expand our vision of dynamic gas exchange just as rapid arterial pressure transducers opened the way to examine dynamic pulse waveforms. The challenge now is to allow our understanding of the pathophysiology to catch up with the state of the art toolbox.

Neither author has any competing interests or conflicts of interest in relation to this work.
